# Glucocorticoids in Freshwaters: Degradation by Solar Light and Environmental Toxicity of the Photoproducts

**DOI:** 10.3390/ijerph17238717

**Published:** 2020-11-24

**Authors:** Alice Cantalupi, Federica Maraschi, Luca Pretali, Angelo Albini, Stefania Nicolis, Elida Nora Ferri, Antonella Profumo, Andrea Speltini, Michela Sturini

**Affiliations:** 1 Department of Chemistry, University of Pavia, via Taramelli 12, 27100 Pavia, Italy; alice.cantalupi01@universitadipavia.it (A.C.); federica.maraschi@unipv.it (F.M.); luca.pretali@gmail.com (L.P.); angelo.albini@unipv.it (A.A.); stefania.nicolis@unipv.it (S.N.); antonella.profumo@unipv.it (A.P.); 2 Department of Pharmacy and Biotechnology, University of Bologna, via S. Donato 15, 40127 Bologna, Italy; elidanora.ferri@unibo.it; 3 Department of Drug Sciences, University of Pavia, via Taramelli 12, 27100 Pavia, Italy; andrea.speltini@unipv.it

**Keywords:** glucocorticoids, solar light degradation, freshwater pollution, biotoxicity tests

## Abstract

The photodegradation process of seven glucocorticoids (GCs), cortisone (CORT), hydrocortisone (HCORT), betamethasone (BETA), dexamethasone (DEXA), prednisone (PRED), prednisolone (PREDLO) and triamcinolone (TRIAM) was studied in tap and river water at a concentration close to the environmental ones. All drugs underwent sunlight degradation according to a pseudo-first-order decay. The kinetic constants ranged from 0.00082 min^−1^ for CORT to 0.024 min^−1^ for PRED and PREDLO. The photo-generated products were identified by high-pressure liquid chromatography coupled to electrospray ionization tandem mass spectrometry (HPLC-ESI-MS/MS). The main steps of the degradation pathways were the oxidative cleavage of the chain 17 for CORT, HCORT and the rearrangement of the cyclohexadiene moiety for the other GCs. The acute and chronic toxicity of GCs and of their photoproducts was assessed by the *V. fischeri* and *P.* *subcapitata* inhibition assays. The bioassays revealed no significant differences in toxicity between the parent compounds and their photoproducts, but the two organisms showed different responses. All samples produced a moderate acute toxic effect on *V. fisheri* and no one in the chronic tests. On the contrary, evident hormesis or eutrophic effect was produced on the algae, especially for long-term contact.

## 1. Introduction

Glucocorticoids (GCs), the synthetic derivatives of the natural steroid hormone hydrocortisone (HCORT), are the most widely used anti-inflammatory drugs both in human and veterinary therapy [1]. They help to regulate physiological functions such as energy metabolism, immune system response, skeletal growth, cardiovascular function, reproduction, cognition and stress adaption in the vertebrates [2].

Recent literature reports that the total GCs prescriptions largely exceed those of estrogens and androgens due to their significant therapeutic advantages. The efforts made for enhancing GCs activity and bioavailability in the human body have increased their chemical stability [3,4]. Once administered, GCs undergo a partial degradation by metabolic reactions and then a significant part of them, excreted by kidneys [5], reaches unmodified urban wastewater sewage treatment plants (WWTPs), along with many other pharmaceuticals. WWTPs processes cannot adequately treat these components through flocculation or disinfection, and these chemicals are regularly released into the aquatic environment [3,6,7].

The presence of these compounds, even at trace levels, poses a severe threat to the reproduction, growth and development of aquatic organisms [3,4]. A recent study reported that the GCs biodegradability strongly depends on the molecular structure. HCORT and prednisolone (PREDLO) seem to be more sensitive to biological wastewater treatment. Betamethasone (BETA) and its derivatives are moderately biodegradable (ten-fold lower), while the majority of GCs, especially fluocinolone and triamcinolone acetonides (TRIAM), are definitely recalcitrant (up to ten thousand-fold lower) [4]. Moreover, the biotransformation products may maintain a residual endocrine activity as the parent compound, which adds further concern [4].

GCs can be found worldwide from sub-ng L^−1^ up to some hundreds of ng L^−1^ in waters and wastewater effluents. For example, in Breda (Netherlands), prednisone (PRED) and TRIAM were detected in hospital wastewater at 117–545 ng L^−1^ and 14–41 ng L^−1^, respectively [8]. In the southwest of the US, total GC levels ranged between 50 and 90 ng L^−1^ in a WWTP secondary effluent and up to 744 ng L^−1^ in WWTP influents [9,10]. Both natural and synthetic GCs were detected from less than one ng L^−1^ up to 433 ng L^−1^ (cortisone, CORT) in river samples in Beijing and coastal waters of South China [11,12,13].

Over the last few years, many ecotoxicological tests have been carried out regarding the most used drugs, GCs included [14]. Dexamethasone (DEXA), PRED and TRIAM (5–50 nM) produced an alteration in seven genes associated with biological functions in zebrafish larvae [15]. CORT (1–10 µg L^−1^) induced other endocrine-related expressional changes on Zebrafish embryos [16], whereas PRED (0.1–1 µg L^−1^) caused morphological changes, alteration in the skeletal muscle development and precocious hatching [17]. Finally, PREDLO (25 μM) affected the hypothalamic–pituitary–interrenal axis activity [18]. GCs in mixtures showed additive activity [19].

Although it has been demonstrated that solar light is an effective disinfection method [20,21], to our knowledge, the environmental fate of GCs and of their products formed during the degradation process, as well as their impact on water quality, have not yet been examined under actual conditions, viz. low-level concentrations, natural waters and sunlight irradiation. GCs degradation was studied under experimental conditions different from the above-mentioned ones. DellaGreca et al. [22,23] investigated the photolysis of DEXA, PRED and PREDLO aqueous suspensions under simulated solar light and visible light. However, they irradiated a concentration (200 mg L^−1^) much larger than the compounds’ solubility limit in order to isolate and characterize the photoproducts by NMR, and thus their results concerned the photochemistry in the solid-state. More recently, Cacciari et al. [24] studied the photophysical and photochemical behavior of PREDLO (14.4 mg L^−1^) in a water-acetonitrile mixture under UV-B light to evaluate the presence of reactive oxygen species involved in the process.

**Figure 1:**
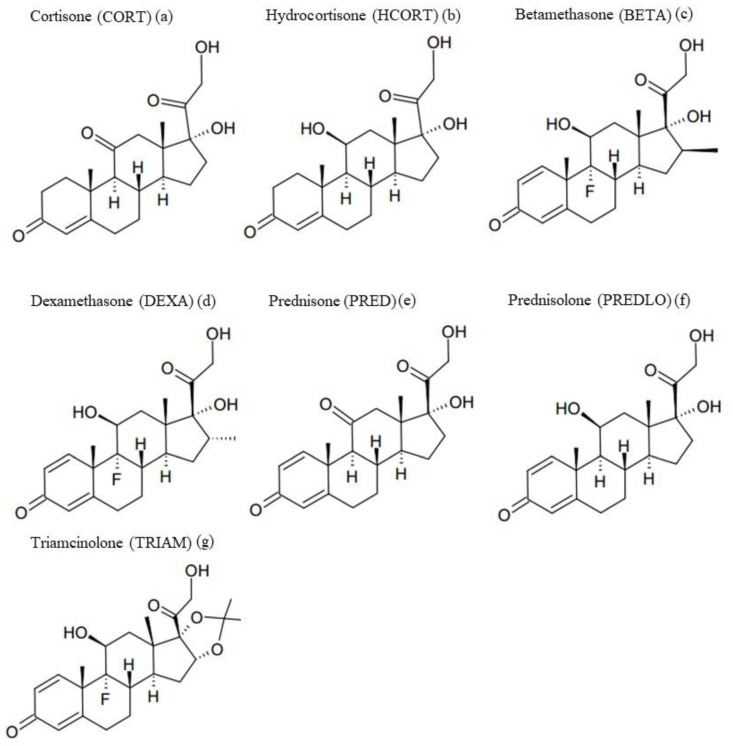
Molecular structures of the investigated glucocorticoids (GCs). (**a**): cortisone; (**b**): hydrocortisone, (**c**): betamethasone, (**d**): dexamethasone, (**e**): prednisone, (**f**): prednisolone and (**g**): triamcinolone.

We considered worthwhile to carry out an in-depth exploratory study on a range of GCs. The chosen drugs are shown in Figure 1.

The photodegradation was performed, both in tap and river water, at 50 µg L^−1^ concentration to mimic and easily follow the oxidation process under natural conditions. Moreover, 10 mg L^−1^ tap water solution was employed in order to obtain an amount of photoproducts sufficient for their good characterization by HPLC-ESI-MS/MS and elucidation of the photochemical path. The evaluation of the biotoxicity of irradiated and non-irradiated drug solutions—the first test to evaluate the environmental impact of xenobiotics—was performed by applying two largely used ISO standard biological tests for water quality assessment based on *Vibrio fischeri* light emission inhibition and *Pseudokirchneriella subcapitata* algal growth inhibition, respectively (ISO 11348-3:2007; ISO 14442:2006) [25,26].

## 2. Materials and Methods

### 2.1. Reagents and Materials

Analytical-grade GCs standards (CORT, HCORT, BETA, DEXA, PRED and PREDLO), acetic acid (99–100%) and acetonitrile (ACN) were purchased by Sigma-Aldrich (Milan, Italy). Analytical-grade TRIAM was supplied by Farmabios (Gropello Cairoli, Italy). HPLC gradient-grade methanol (MeOH) and ultrapure water were purchased by VWR (Milan, Italy). GCs stock solutions of 10 mg L^−1^ were prepared in tap water and stored in the dark at 4 °C for a maximum of a week. Working solutions of 50 µg L^−1^ were prepared daily.

Lyophilized aliquots of the luminescent bacteria *V. fischeri* were prepared from fresh cultures at our laboratory, starting from an original batch supplied by the Pasteur Institute (Paris, France). Thermo Scientific (Vantaa, Finland) supplied the 96-well “Black Cliniplate” microplates. Nutrient broth components were obtained from Sigma-Aldrich (Table S1). The Istituto Zooprofilattico Sperimentale of Abruzzo and Molise “G. Caporale” (Teramo, Italy) supplied the freshwater microalgae *P. subcapitata* culture. Inorganic salts and nutrients for algal growth were obtained from Sigma-Aldrich (Table S2).

(Chlorinated) tap water, chosen because of its invariant composition and greater similarity to natural waters than the ultrapure one, was from the Pavia municipal waterworks. Freshwater from the Staffora River was collected at 30–50 cm-depth in amber glass bottles. All the samples were stored in the dark (4 °C) before use. The physicochemical parameters are reported in Table 1.

### 2.2. Irradiation Experiments

Different irradiation experiments were carried out in order to: (1) obtain the degradation profiles at two concentrations (50 µg L^−1^ and 10 mg L^−1^), (2) identify the irradiation time corresponding to the maximum concentrations of generated photoproducts and (3) prepare the solutions to perform the toxicity tests on GCs photoproducts [27,28,29,30] (and reference herein).

#### 2.2.1. Kinetic Profiles at 50 µg L^−1^ Concentration

100 mL of tap and river water samples were enriched with 50 µg L^−1^ of anti-inflammatory drugs, each of them separately dissolved. In a closed glass container (depth 40 mm, exposed surface 9500 mm^2^), each solution was irradiated by a solar simulator (Solar box 1500e, CO.FO.ME.GRA, Milano, Italy) set at a power factor 250 W m^−2^, equipped with a UV outdoor filter of soda-lime glass IR treated and with a BST temperature sensor. Aliquots (0.5 mL) of each sample were withdrawn at planned times, filtered (0.22 µm) and injected in the HPLC-ESI-MS/MS system (multiple reaction monitoring mode, MRM).

#### 2.2.2. Kinetic Profiles at 10 mg L^−1^ Concentration and Identification of the Photoproducts

100 mL tap water samples were spiked with 10 mg L^−1^ of GCs (each of them separately dissolved) and irradiated as described before. Aliquots (0.5 mL), treated as above, were injected in the HPLC-UV system prior to performing the HPLC-ESI-MS/MS analysis (in full scan, zoom scan and MS/MS mode) for the identification of the photoproducts.

Each kinetic experiment was performed in triplicate, and the degradation kinetic constant (*k_deg_*) was calculated by using a dedicated software (Fig P application, Fig P Software Corporation, version 2.2a, BIOSOFT, Cambridge, UK).

### 2.3. Analytical Determinations

Different HPLC systems with different sensitivity (instrumental quantification limits, IQLs) and different modes of analysis were employed to quantify and identify the GCs’ photoproducts at the two working concentrations [27,28,29,30] (and reference herein).

#### 2.3.1. Kinetic Profiles at 50 µg L^−1^ Concentration

An Agilent (Cernusco sul Naviglio, Italy) HPLC 1260 Infinity apparatus coupled with an Agilent 6460C ESI-MS/MS spectrometer was used. Each sample (5 µL) was injected into an Agilent 120 EC-C18 Poroshell column (50 × 3 mm, 2.7 µm) with a similar guard-column. Ultrapure water (0.1% *v/v* acetic acid) (A) and MeOH (0.1% *v/v* acetic acid) (B) were used as mobile phases. A linear gradient from 40% to 84% B was applied in 12 min, followed by a column re-equilibration time of 8 min (0.6 mL min^−1^ flow rate, column temperature 50 ± 1 °C).

The MS/MS system consisted of a triple quadrupole with an electrospray ionization (ESI) source operating in negative mode (precursor ion [M + AcO]^−^ adduct). The source parameters were: drying gas temperature 300 °C (N_2_); drying gas flow 5 L min^−1^ (N_2_); nebulizer 45 psi; sheath gas temperature 250 °C; sheath gas flow 11 L min^−1^; capillary voltage 3500 V (positive mode) and 3000 V (negative mode); nozzle voltage 500 V positive, 0 V negative; electron multiplier voltage (EMV) 0 V for both polarities; cell accelerator voltage (CAV) 1 V. The optimized multiple reaction monitoring (MRM) conditions for the target analytes are reported in Table S3. The instrumental quantification limits were in the range of 0.2–0.6 μg L^−1^. The MassHunter software from Agilent was used for data processing.

#### 2.3.2. Kinetic Profiles at 10 mg L^−1^ Concentration

The HPLC-UV system consisted of a Shimadzu (Shimadzu Corporation, Milano, Italy) LC-20AT solvent delivery module equipped with a DGU-20A3 degasser and interfaced with an SPD-20A UV detector. The wavelength selected for analysis was 238 nm that is included in the absorption band of all the GCs. Each sample was diluted (30% *v*/*v*) with MeOH and injected (20 µL) into a 250 × 4.6 mm, 5 µm GraceSmart RP18 (Sepachrom) column, coupled with a similar guard-column. Isocratic elution was carried out for 20 min using ultrapure water–ACN mixture, 70:30 for CORT, HCORT, PRED and PREDLO; 65:35 for BETA, DEXA and TRIAM. After washing for 5 min with 100% ACN, the initial conditions were reestablished. The flow rate was 1.0 mL min^−1^. The instrumental quantification limits were 0.3 mg L^−1^ for CORT, HCORT and DEXA, 0.09 mg L^−1^ for BETA, 0.2 mg L^−1^ for PRED, PREDLO and TRIAM.

#### 2.3.3. Identification of the photoproducts

The HPLC-ESI-MS/MS analyses for photoproducts identification were performed by using a surveyor HPLC system (Thermo Finnigan, San Jose, CA, USA), equipped with a 150 × 2.0 mm, 4 µm Jupiter 4U Proteo column (Phenomenex). The mobile phase consisted of ultrapure water (0.1% *v/v* formic acid) (A) and ACN (0.1% *v/v* formic acid) (B). The starting concentration of eluent B was 2%, increased to 100% by 40 min with a linear gradient. This concentration was maintained for 5 min to wash the column. The flow rate was 0.2 mL min^−1^. The MS/MS system consisted of an LCQ ADV MAX ion-trap mass spectrometer with an ESI ion source operating in ion-positive mode with the following instrument conditions: source voltage 5.0 kV; capillary voltage 46 V; capillary temperature 210 °C; tube lens voltage 55 V. The Xcalibur 2.0.7 SP1 software (Thermo Finnigan, San Jose, CA, USA) was used for spectra processing.

### 2.4. Toxicity Assays

Samples for the biotoxicity tests were prepared, starting from the 10 mg L^−1^ tap water solutions of each GC.

Bacteria and algal growth inhibition tests were determined in parallel for non-irradiated A solutions and irradiated B solutions of each drug.

The A-type samples contained the same amount of the parent compound measured in the B-type as a residue after irradiation. They were prepared by dilution of the 10 mg L^−1^ solutions. The B-type samples contained the maximum amount of products generated during the irradiation process, as verified by HPLC-UV and a residue of the parent compound.

In addition, for each GC, a series of solutions in the range of 0.5–10 mg L^−1^ were prepared by diluting the 10 mg L^−1^ solutions to evaluate the EC_50_ values for bioluminescence bacteria after chronic exposure (24 h).

#### 2.4.1. Bioluminescence Inhibition Assay

We reconstituted lyophilized aliquots of *V. fischeri* containing NaCl 3% *w/v* by adding 1 mL of distilled water and resuspended them in 10–30 mL of the nutrient broth (see Table S1). Two hundred microliters of the bacteria suspension and 100 µL of each sample were added to the microplate wells. The controls consisted of 200 µL of the bacteria plus 100 µL of a 3% NaCl solution in tap water. The emitted light was recorded by a Victor Light 1420 microplate luminometer (Perkin-Elmer, Norwalk, Conn. USA) at fixed intervals between 0 and 48 h. Five replicates were prepared for each sample, and the light emission values expressed as relative luminescence units (RLU).

The bioluminescence inhibition percentage (*I*%) was used to express the toxicity of the tested samples (A and B types) and calculated according to:

(1) $$I\%\;=\;\frac{L_{blank}-\;L_{sample}}{L_{blank}}\;\times 100$$

where *L* is the intensity of the light emitted by the sample or by the control (blank).

#### 2.4.2. Algal Growth Inhibition Assay

The starter culture of *P. subcapitata* was prepared by inoculating 1 mL of microalgae suspension on an Erlenmeyer flask containing 100 mL of the Jaworski’s culture medium (see Table S2). The flasks were stopped by a porous cotton plug and illuminated by a white lamp/red lamp Osram daylight 2 × 36 W plus Osram Gro-Lux lamp 36 W, (8 h light/16 h dark) at 20 °C.

To perform the assay, smaller flasks were filled with 20 mL of a dilution of the logarithmic phase algae culture (approximately 10^5^ cells mL^−1^). To the irradiated or non-irradiated GC solutions, the appropriate amount of the Jaworski’s salts mixture was added, and then 10 mL transferred to the flask. The small flasks were kept in the same conditions as the starter culture. Controls were prepared by adding 10 mL of the Jaworski’s salts mixture to 20 mL of algae suspension.

We evaluated the algal density by measuring the absorbance at λ 684 nm, an indirect method for cell counting also mentioned in the ISO 8692/2004.

Each sample was tested in triplicate.

## 3. Results and Discussion

### 3.1. Photolysis of GCs in Environmental Samples

The behavior of the most used steroids (see Figure 1) was studied under realistic and well-controlled conditions.

A series of irradiation experiments were performed on tap and not filtered river water samples spiked with 50 μg L^−1^ of each GC (separately dissolved) under simulated solar light to mimic the real environmental conditions and to follow the degradation process easily.

**Figure 2:**
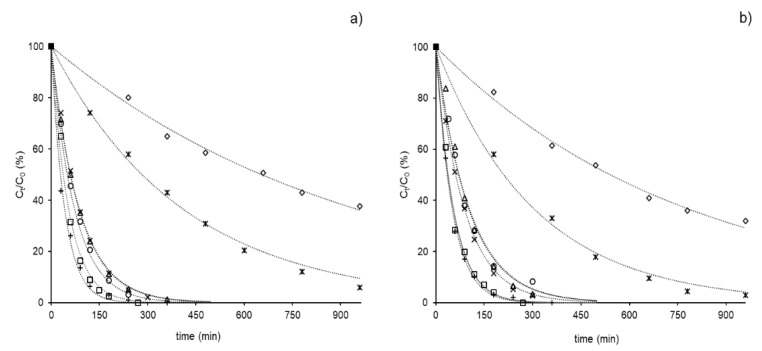
Evolution profiles of each GC (50 µg L^−1^) both in tap (**a**) and river water (**b**) under simulated sunlight cortisone (CORT) (◊), hydrocortisone (HCORT) (✴), betamethasone (BETA) (×), dexamethasone (DEXA) (∆), prednisone (PRED (☐), prednisolone (PREDLO) (+) and triamcinolone (TRIAM) (○) (see Section 2.2. for the irradiation conditions).

As shown in Figure 2a,b, all drugs proved to be susceptible to photodegradation under simulated outdoor conditions, although the quantum yields, as previously observed, were very low [31].

The experimental data fit well a pseudo-first-order equation:

(2) $$\frac{C_{t}}{C_{0}}=\;e^{-k_{deg}t}$$

where *C*_0_ is the initial GC concentration, *C*_t_ is the GC concentration at time *t*, and *k_deg_* is the kinetic degradation constant.

The kinetic degradation constants (*k_deg_*) were reported in Table 2, along with those obtained in tap water at 10 mg L^−1^. As confirmed by *k_deg_* values, no difference in the order of reactivity was observed at a higher GCs concentration.

In details, in tap water (Figure 2a), PRED and PREDLO were fully degraded in about 3 h, BETA, DEXA and TRIAM in about 5 h, while CORT and HCORT decreased more slowly, and about 38 and 6% of the initial amounts, respectively, were still present after 16 h of exposure.

In river water samples (Figure 2b), similar profiles were observed. In particular, PRED and BETA showed the same trend as in tap water, while CORT and HCORT decomposed slightly faster and PREDLO, DEXA and TRIAM slightly lower than in tap water.

Due to the small differences in *k_deg_* values and the similar composition of the two matrices (see Table 1), we excluded a specific contribution to the photodegradation rate by the matrix constituents. Ubiquitous species, such as natural organic matter (NOM) that often affect the photodegradation process [32,33], especially in environmental conditions, seemed to have a negligible effect at the concentration present in our samples. However, it is possible to assume that NOM contributed to the conversion of the less light-absorbing drugs (simply conjugated ketones) CORT and HCORT, whereas, when more light-absorbing (cross-conjugated ketones) drugs were present, NOM competed for a significant fraction of light. In both matrices, *k_deg_* values for CORT and HCORT were one order of magnitude lower than those calculated for the other GCs.

No decomposition was observed in aqueous solutions fortified with 50 μg L^−1^ of each GCs and kept in the dark at room temperature for a monitoring period of 3 h for PRED and PREDLO, 5 h for BETA, DEXA and TRIAM and 16 h for CORT and HCORT (see Figure S1).

Further experiments were carried out at 10 mg L^−1^ concentration (close to the maximum GCs water solubility) in tap water to obtain a sufficiently large amount of the photoproducts to facilitate both HPLC-MS characterization and biotoxicity tests (see Figure S2). Table 3 shows, for each GC, the irradiation time corresponding to the maximum concentration of photoproducts generated during the irradiation process, the residual GCs concentration at that time and the percentage of GCs degradation. By the way, the reported residual concentrations of the parent compound are the GCs concentrations in the A-type samples (GCs at the same concentration of B-type samples and not containing photoproducts).

**Scheme 1:**
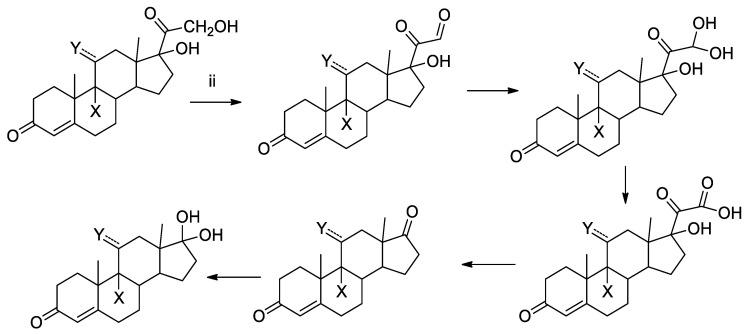
Main reaction path of compounds **a**,**b**.

**Scheme 2:**
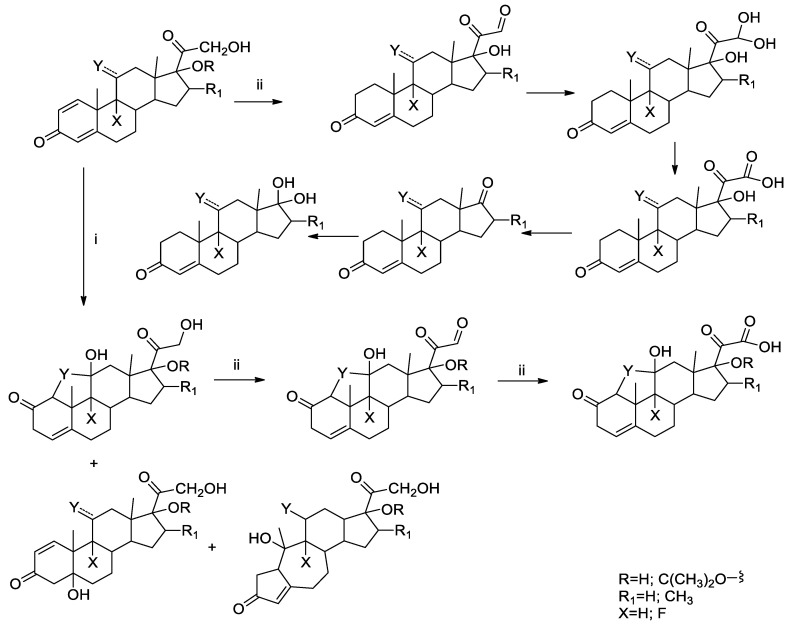
Main reaction path of compounds **c**–**g**, according to the paths i and ii.

The generated photoproducts, present to the extent of a few percent with respect to the initial amount of each GC, were characterized by HPLC-ESI-MS/MS and allowed to elucidate the general mechanistic degradation pathway (see Scheme 1 and Scheme 2 and Tables S4 and S10).

Two main paths were observed in the photochemical reaction of compounds **a**–**g**. The first (i) corresponds to the isomerization of the cyclohexadienone moiety (**c**–**g**) and the second one (ii) to the oxidative cleavage of the chain in 17 for all the compounds. Path (ii) is also the main photodegradation route for **a**,**b**, for which path (i) is not available.

When no isomerizable moiety is present, the main reactivity site of compounds **a**,**b** is the hydroxyl acetone moiety in position 17. As a consequence, some products were identified (see Scheme 1), and the structures were proposed based on their MS/MS fragmentation pattern (see Tables S4 and S5) and in accordance with literature data [34,35]. The subsequent oxidation-hydration steps (ii) proceed via a series of derivatives, well in accordance with the stepwise conversion to aldehyde, acetal, carboxylic acid, ketone and ketal.

On the contrary, the second conjugated double-bound on ring A opens up another primary route for compounds **c**–**g**, i.e., the cyclohexadienone isomerization (i), while pathway (ii) from the parent compound remains as a minor reaction (see MS/MS fragmentation pattern, Tables S6–S10). Through pathway (i), two different rearrangement routes lead either to a contraction/expansion of the A and B rings, respectively or to a ring closure between C1 and C11 with the formation of a new condensed tetrahydrofuran moiety. Both these rearrangements proceed via a common lumi-derivative intermediate [36,37,38,39,40]. This class of compounds may then further react through the path (ii) leading to C17 side-chain oxidation products.

The chemistry observed in the aqueous matrix well matches that previously reported in the literature in the presence of organic solvents [36,40].

### 3.2. Ecotoxicity of GCs Photoproducts

The *V. fischeri* test was selected because of its widespread application in monitoring and quality control activities on surface and seawater bodies [41]. *V. fischeri* is a strain of marine luminescent bacteria, often living as symbionts in the luminescent organs of various marine organisms. The light emission phenomenon requires the presence of oxygen, which oxidizes FMNH_2_ and a long-chain aldehyde under the enzymatic catalysis of a Luciferase. It is an energy-consuming process, and it occurs only in cells, other conditions being the same, in optimal structural and metabolic state. In case of appearance in their environment of something affecting this state, for example, a toxic compound, the bacteria suppress or reduce the light emission accordingly to the intensity of the damage produced by this compound. The tight correlation between the biotoxicity of a xenobiotic and the intensity of light emission was clearly demonstrated, and this assay becomes an ISO reference test widely accepted as a rapid tool to define the water quality (ISO 11348-3:2007). A strong reduction (inhibition) of the light emissions means the presence of a highly toxic contaminant [25,41].

In a similar way, the growth of any aquatic organism can be impaired in not optimal environmental conditions, i.e., a reduction in the reproduction rate indicates the presence of toxic xenobiotics or an extreme alteration in physical parameters. The microalgae are at the basis of the food chain, and *P. subcapitata* was chosen to set up another water quality ISO assay because this green microalga is representative both of eutrophic and oligotrophic freshwater environments [26].

A correct evaluation of organic pollutants ecotoxicity must take into account the contribution of both the parent compounds and of the photoproducts since these could preserve the parental activity and/or contribute to the appearance of different ecotoxic effects [29]. Moreover, performing the treatment of pollutants whose toxicity is lower than their treatment derivatives would clearly be of negative value.

In this work, the potential additive/synergistic toxic contribution of the photoproducts to the environmental toxicity of each parental GC was evaluated by comparing the intensity of the emitted light and the algal growth rate in the presence of the B-type samples with those measured in the presence of the respective A-type samples.

In addition, the original 10 mg L^−1^ GCs solutions, diluted in the range 10–0.5 mg L^−1^, were tested on bioluminescence bacteria in the same conditions of other samples in order to obtain the EC_50_ values after chronic exposure (24 h).

For the most part of the compounds, the 10 mg L^−1^ samples were not able to reduce to 50% the light emission. The EC_50_ of TRIAM was just 10 mg L^−1^, DEXA showed an EC_50_ equal to 8 mg L^−1^ and the highest toxicity was attributed to CORT, with an EC_50_ equal to 6 mg L^−1^.

**Figure 3:**
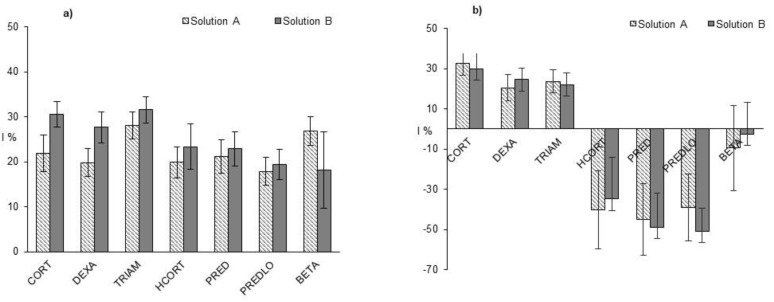
Bioluminescence inhibition (*I*%) recorded for solution B (gray bars) and solution A (stripped bars) after 5 h (**a**) and 24 h contact (**b**). Error bars represent the standard deviation of *I*%, obtained by means of error propagation calculation.

Figure 3 shows the % light inhibition data obtained for the A and B-type samples after 5 and 24 h contact with *V. fischeri*.

After 5 h contact, the observed bioluminescence inhibition values were the same for the irradiated (B) and non-irradiated (A) samples (two-way ANOVA test, *p* = 0.05).

After 24 h contact, there is still no difference between (B) and (A) samples, while it appeared a highly significant difference among the studied GCs (two-way ANOVA test, *p* = 0.05). It was evident that HCORT, PRED and PREDLO produced a eutrophic effect, demonstrated by the negative values of the bioluminescence inhibition (*I*%), while CORT, DEXA and TRIAM still had an inhibition effect. We attempted to propose a rationalization of these effects even though, as far as we know, no generally valid toxicity-structure relationship has been established yet for GCs [42]. The double conjugated ketones PRED and PREDLO, which are good radical traps, and HCORT, the best H-donor among the tested GCs, show a eutrophic effect. On the contrary, all the fluorine bearing GCs (TRIAM, DEXA) show a moderate toxic effect and CORT, a single conjugated GC, shows the highest toxic effect. Only for BETA, it was not possible to identify a clear effect since *I*% was around zero.

**Figure 4:**
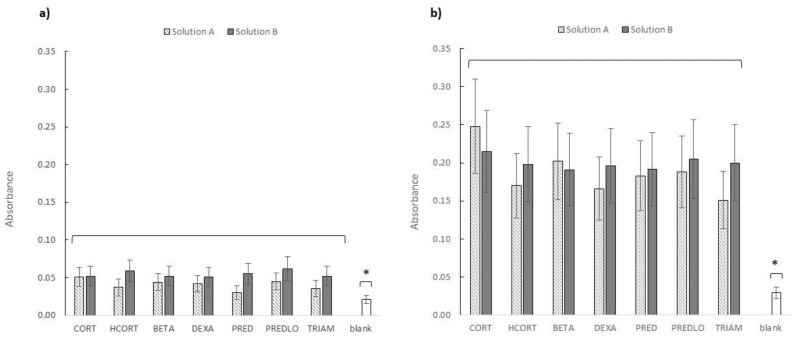
Absorbance recorded at λ = 648 nm for green algae suspension in contact with GCs samples B (gray bars) and A (stripped bars) after 8 (**a**) and 15 (**b**) days contact. Error bars represent the standard deviation of three independent experiments. The asterisk indicates a statistically significant difference between the sample absorbance mean (samples A and B) and the blank sample absorbance (confidence level *p* = 0.05).

The growth rate of the green microalga *P. subcapitata* was determined by measuring the suspension’s absorption. After 8 d contact time, it was possible to affirm that the absorbance observed in the presence of the irradiated (B) samples were different from those in the presence of the (A) samples, being the ***F****_sper_* value above the critical one (two-way ANOVA test, *p* = 0.05). Concerning the samples at 15 d contact time, the slight difference previously observed disappeared (***F****_sper_* values were always below the critical ones, at *p* = 0.05). This effect could be the result of increased variability in the measures. Nevertheless, at any contact time was possible to observe the complete absence of any growth inhibition effect on the microalgae cultures in the presence of both types of solutions, as shown in Figure 4.

More exactly, a growth-stimulating effect was evident, i.e., the absorbance values increase as the contact time increases. The difference between the mean absorbance values of samples A and B and that of the control (blank) was the clearest proof. For the confidence level at *p* = 0.05, the confidence intervals for the irradiated and non-irradiated values were calculated, and in both cases, the blank value was outside the interval and significantly lower.

Similar effects have been already observed on two different algal species, *M. flos-aquae* and *S. obliquus*, in the presence of DEXA solutions before and after gamma irradiation [43]. The *M. flos-aquae* and *S*. *obliquus* growth increased by increasing the time of contact and the GC concentrations both in the presence of irradiated and non-irradiated solutions.

On the other hand, DEXA, PRED and PREDLO and their photoproducts, tested by Della Greca et al. [22,23], exerted a negligible effect on *P. subcapitata* growth rate.

The most probable explanation for the absence of the toxic effect of the GCs on the algae is the completely different metabolism of these vegetable cells with respect to animal organisms. At first glance, this should be considered positive information, but a very negative one is the undeniable eutrophic stimulus produced by the presence of these compounds. GCs and their photoproducts can contribute, together with an endless list of other organic compounds, to the occurrence of algal blooms, which we know to be a serious adverse effect in natural environments.

## 4. Conclusions

In this explorative study, the sunlight degradation of seven widely used GC was compared in tap and river waters. The photodegradation process occurs, although it may be slower than for other classes of drugs, also in the case of CORT and HCORT, which scarcely absorb solar light. Sunlight photolysis was confirmed to be a valid abiotic removal pathway for alleviating the accumulation of such persistent xenobiotics into the aquatic systems. The various photoproducts formed during the photolytic process were identified, and this allowed elucidating the general mechanistic degradation pathway of this class of compounds, i.e., the oxidative cleavage of the chain 17 for CORT and HCORT and the rearrangement of the cyclohexadienone moiety for the other GCs.

Two living organisms, *V. fischeri* and *P. subcapitata,* were used to evaluate the potential ecotoxic effects due to the presence of GCs and their photoproducts in the environment. Both the assays showed useful information. The first one demonstrated a moderate ecotoxic effect for both irradiated and non-irradiated GCs solutions at a short-term contact time and a eutrophic one for both irradiated and non-irradiated HCORT, PRED and PREDLO solutions at 24 h contact. A proposal for this behavior resulting from the intervention of radical paths was tentatively proposed.

Algal growth inhibition test, always representing a chronic exposure test, showed a eutrophic or hormesis effect, which increased for the long-term assay in the presence of both irradiated and non-irradiated GCs solutions, confirming data previously reported in the literature under oxidative conditions.

The reported results further showed the great complexity of environmental effects produced by persistent organic pollutants, both unmodified and degraded through natural processes. Both the eutrophic and toxic effects can lead to environmental unbalance. It would be useful, in the future, to clarify how different or higher content of matrix constituents may affect the oxidative process involved with GCs and, in general, with the solar disinfection efficiency.

## Figures and Tables

**Table 1:** Physicochemical characterization of tap and river water samples.

Parameters/Ions		Tap Water	River Water
*pH*		7.7	7.9
*Conductivity at 20 °C*	μS cm^−1^	271	293
*Total organic carbon (TOC)*	mg L^−1^	4.2	7.0
*Cl^−^*	mg L^−1^	5.0	4.0
*NO_3_^−^*	mg L^−1^	0.6	1.6
*SO_4_^2−^*	mg L^−1^	5.0	13
*HCO_3_^−^*	mg L^−1^	182	209
*Ca^2+^*	mg L^−1^	35	54
*Mg^2+^*	mg L^−1^	10	7.8
*Na^+^*	mg L^−1^	12	5.4

**Table 2:** Direct photolysis degradation constants (*k_deg_*) for each GC in tap and river water under simulated sunlight (see Section 2.2 for the irradiation conditions). Cortisone (CORT), hydrocortisone (HCORT), betamethasone (BETA), dexamethasone (DEXA), prednisone (PRED), prednisolone (PREDLO) and triamcinolone (TRIAM).

Compound	10 mg L^−1^	50 µg L^−1^	50 µg L^−1^
	*k_deg_* (min^−1^), Tap Water	*k_deg_* (min^−1^), Tap Water	*k_deg_* (min^−1^), River Water
CORT	0.00082 (8)	0.00106 (5)	0.00128 (3)
HCORT	0.00110 (7)	0.00246 (9)	0.0033 (2)
BETA	0.0133 (3)	0.0115 (6)	0.0114 (1)
DEXA	0.0139 (5)	0.0117 (1)	0.0097 (6)
PRED	0.024 (1)	0.0185 (9)	0.0186 (6)
PREDLO	0.0181 (4)	0.024 (1)	0.0199 (4)
TRIAM	0.0130 (5)	0.0139 (3)	0.0099 (4)

In brackets, the standard deviation values of the least significant digit; R^2^ values in the range 0.992–0.999.

**Table 3:** Irradiation time corresponding to the maximum amount of photoproducts generated during the irradiation process in the presence of the parent compound residue, percentage of GCs degradation.

Compound	*Irradiation Time* (min)	*Conversion* (%)	*Parent Compound Residue* (mg L^−1^)
CORT	1320	73	2.7
HCORT	1320	77	2.3
BETA	90	67	3.2
DEXA	90	69	3.1
PRED	120	93	0.7
PREDLO	30	41	5.9
TRIAM	60	62	3.8

## Supplementary Materials

The following are available online at https://www.mdpi.com/1660-4601/17/23/8717/s1, Table S1: Nutrient broth for bioluminescent bacteria assay. Table S2: Jaworski’s culture medium for algal growth inhibition assay. Table S3: Optimized MRM conditions for the HPLC-ESI-MS/MS analysis. Table S4: Fragmentation of photolytic products of CORT ([M + 1]^+^ = 361). Table S5: Fragmentation of photolytic products of HCORT ([M + 1]^+^ = 363). Table S6: Fragmentation of photolytic products of BETA ([M + 1]^+^ = 393). Table S7: Fragmentation of photolytic products of DEXA ([M + 1]^+^ = 393), Table S8: Fragmentation of photolytic products of PRED ([M + 1]^+^ = 359), Table S9: Fragmentation of photolytic products of PREDLO ([M + 1]^+^ = 361), Table S10: Fragmentation of photolytic products of TRIAM ([M + 1]^+^ = 435). Figure S1: Tap water solutions fortified with 50 µg L−1 of each GCs and kept in the dark at room temperature for a monitoring period of 3 h for PRED (☐) and PREDLO (+), 5 h for BETA (×), DEXA (∆) and TRIAM (○), 16 h for CORT (◊) and HCORT (✴). Figure S2: HPLC-UV chromatogram of BETA (black line) in the presence of the maximum amount of photoproducts (red line) (a); photodegradation profile of BETA and evolution profile of the generated photoproducts verified by HPLC-UV (b) (90 min irradiation).
