# Supplementary material for: Glucocorticoids in Freshwaters: Degradation by Solar Light and Environmental Toxicity of the Photoproducts
Source: Int J Environ Res Public Health. 2020 Nov 24;17(23):8717. doi: 10.3390/ijerph17238717 (PMC7727706; doi:10.3390/ijerph17238717)

Article

Glucocorticoids in Freshwaters: Degradation by Solar Light and Environmental Toxicity of the Photoproducts

Alice Cantalupi [1], Federica Maraschi [1], Luca Pretali [1], Angelo Albini [1], Stefania Nicolis [1], Elida Nora Ferri [2], Antonella Profumo [1], Andrea Speltini [3] and Michela Sturini [1,*]

[1] Department of Chemistry, University of Pavia, via Taramelli 12, 27100 Pavia, Italy; alice.cantalupi01@universitadipavia.it (A.C.); federica.maraschi@unipv.it (F.M.); luca.pretali@gmail.com (L.P.); angelo.albini@unipv.it (A.A.); stefania.nicolis@unipv.it (S.N.); antonella.profumo@unipv.it (A.P.)

[2] Department of Pharmacy and Biotechnology, University of Bologna, via S. Donato 15, 40127 Bologna, Italy; elidanora.ferri@unibo.it

[3] Department of Drug Sciences, University of Pavia, via Taramelli 12, 27100 Pavia, Italy; andrea.speltini@unipv.it

* Correspondence: michela.sturini@unipv.it; Tel.: +39-0382-987347

Received: 20 October 2020; Accepted: 20 November 2020; Published: date

Table S1. NUTRIENT BROTH FOR BIOLUMINESCENT BACTERIA ASSAY (final volume 500 mL, pH=7).

NaCl	g	15
Peptone	g	2.5
Yeast extract	g	1.5
Glycerol	mL	1.5
HEPES	M	0.01

Table S2. JAWORSKI'S CULTURE MEDIUM FOR ALGAL GROWTH INHIBITION ASSAY.

$(Ca(NO_3)_2 \cdot 4H_2O$	g L^{-1}	20
KH_2PO_4	g L^{-1}	12.4
$MgSO_4 \cdot 7H_2O$	g L^{-1}	50
$NaHCO_3$	g L^{-1}	15.9
EDTAFeNa	g L^{-1}	2.25
$EDTANa_2$	g L^{-1}	2.25
H_3BO_3	g L^{-1}	2.48
$[(NH_4)_6Mo_7O_{24} \cdot 4H_2O]$	g L^{-1}	1
$MnCl_2 \cdot 4H_2O$	g L^{-1}	1.4
cyanocobalamin	g L^{-1}	0.04
biotin	g L^{-1}	0.04
thiamine	g L^{-1}	0.04
$NaNO_3$	g L^{-1}	80
$NaH_2PO_4 \cdot 2H_2O$	g L^{-1}	36

Table S3. OPTIMIZED MRM CONDITIONS FOR THE HPLC-ESI-MS/MS ANALYSIS.

Target[a]	Parent peak[b] (m/z)	MRM product ions (m/z)	Dwell time	Fragmentor Energy (V)	Collision Energy (V)
CORT	419	359.4	50	91	0
		329.4	50	91	8
HCORT	421.5	331.4	50	91	12
		297.3	50	91	36
PRED	417	357.3	50	81	0
		327.3	50	81	8
PREDLO	419	329.3	50	116	12
		295.3	50	116	32
BETA	451.5	361.4	50	106	12
		307.3	50	106	32
DEXA	451.5	361.4	50	91	12
		307.3	50	91	32
TRIAM	493.5	413.4	80	133	16
		59	80	133	24

[a] retention time (min): CORT 3.84; H-CORT 4.61; PRED 3.52; PREDLO 4.59; BETA 6.33; DEXA 6.33; TRIAM 6.72. [b] $[M+AcO]^-$ adduct.

Table S4. FRAGMENTATION OF PHOTOLYTIC PRODUCTS OF CORT **(**$[M+1]^+$=361)

Fragment	HPLC-ESI-MS/MS							
	**28,94**		**28,47**		**31,92**		**27,25**	
	m/e	Int%	m/e	Int%	m/e	Int%	m/e	Int%
$[M+1]^+$	361,16	10	377,28	0	375,4	0	379,31	0
$[M+1]^+$-H_2O	343,11	100	359,13	70			361,33	100
$[M+1]^+$-H_4O_2	325,10	70	341,04	100				
$[M+1]^+$-CO_2					330,75	15		
$[M+1]^+$-CH_2O_2	315,10	10						
$[M+1]^+$-CH_4O_2	313,07	22						
$[M+1]^+$-H_6O_3	307,09	40	323,13	30				
$[M+1]^+$-CH_4O_3			313,19	20				
$[M+1]^+$-C_2H_6O								
$[M+1]^+$-C_3H_2O								
$[M+1]^+$-C_3H_6+H_2O								
$[M+1]^+$-C_5H_4								

[M+1]+-C3H6O								
[M+1]+-C2H6O2	299,10	38						
[M+1]+-CH4O3					311,16	20		
[M+1]+-CH6O3	295,12	24						
[M+1]+-C2H2O3					301,06	100		
[M+1]+-C2H4O3	285,06	26						
[M+1]+-C3H8O2								
[M+1]+-C2H6O3	283,11	40	299,08	22				
[M+1]+-C3H8O3					283,37	100		
[M+1]+-CH6O4			295,14	40				
[M+1]+-C2H8O4			281,05	22				
[M+1]+-C2H10O3	279,12	22						
[M+1]+-C2H6O4	267,10	38						
[M+1]+-C6H6O2					265,18	80		
[M+1]+-C2H8O4	265,11	34						
[M+1]+-C2H12O5							281,28	30
[M+1]+-C9H6								
[M+1]+-C8H6O								
[M+1]+-C12H13								
[M+1]+-C10H12O2							215,48	60

Table S5. FRAGMENTATION OF PHOTOLYTIC PRODUCTS OF HCORT ([M+1]+=363)

Fragment	HPLC-ESI-MS/MS									
	**32,13**		**28,91**		**32,11**		**28,02**		**29,31**	
	m/e	int%	m/e	Int%	m/e	Int%	m/e	Int%	m/e	Int%
[M+1]+	303,2	0	362,9	1	377,3	0%	379,2	2	361,1	4
[M+1]+-CH3					363,08	10				
[M+1]+-OH										
[M+1]+-H2O	285,06	100	345,17	40			361,12	10	343,07	100
[M+1]+-H3O2										
[M+1]+-H4O2	267,13	60	327,12	100			343,11	100	325,06	70
[M+1]+-CH2O2	256,95	20			331,11	24				
[M+1]+-H6O3	249,21	20	309,13	80			325,09	70	307,11	52
[M+1]+-CH2O3									299,06	40
[M+1]+-CH4O3					3131,4	20	315,16	22		
[M+1]+-H8O4							307,11	28		
[M+1]+-CH6O3			297,11	30						

$[M+1]^+$-CH_3O_4	239,07	40								
$[M+1]^+$-CH_6O_4			281,09	28			297,09	62		
$[M+1]^+$-$H_{10}O_5$							289,14	10		
$[M+1]^+$-$C_2H_6O_3$									283,07	40
$[M+1]^+$-$C_2H_5O_4$	225,09	10								
$[M+1]^+$-$C_2H_8O_4$			267,12	30						
$[M+1]^+$-$C_2H_8O_5$			251,16	12					265,1	44
$[M+1]^+$-$C_3H_4O_2$										
$[M+1]^+$-$C_3H_7O_4$	211,08	6								
$[M+1]^+$-$C_3H_8O_4$							283,12	30		
$[M+1]^+$-$H_{12}O_6$							279,17	28		
$[M+1]^+$-$C_3H_6O_6$									239,11	20
$[M+1]^+$-$CH_{14}O_6$							265,14	6		
$[M+1]^+$-$C_2H_{16}O_6$							251,12	12		
$[M+1]^+$-$C_3H_{18}O_6$							237,18	8		
$[M+1]^+$-$C_4H_{20}O_6$							223,10	12		
$[M+1]^+$-C_5H_4O										
$[M+1]^+$-C_5H_6O					295,07	22				
$[M+1]^+$-$C_5H_7O_2$										
$[M+1]^+$-$C_7H_{10}O$					267,10	100				

Table S6. FRAGMENTATION OF PHOTOLYTIC PRODUCTS OF BETA **(**$[M+1]^+$=393)

Fragment	HPLC-ESI-MS/MS					
	**30,8**		**30,0**		**26,7**	
	m/e	Int%	m/e	Int%	m/e	Int%
$[M+1]^+$	392,90	0	407,33	0	411,37	0
$[M+1]^+$-HF	373,03	100			393,50	20
$[M+1]^+$-CH_2O					381,37	100
$[M+1]^+$-H_3FO	355,10	50				
$[M+1]^+$-H_5FO_2	337,09	20				
$[M+1]^+$-$CHFO_2$			343,41	20		
$[M+1]^+$-CH_3FO_2			340,99	80		
$[M+1]^+$-H_7FO_3	319,12	12				
$[M+1]^+$-$C_3H_6O_2$					337,17	50
$[M+1]^+$-$C_2H_2O_3$			333,23	100		
$[M+1]^+$-$C_2H_7FO_4$	279,14	10				

Table S7. FRAGMENTATION OF PHOTOLYTIC PRODUCTS OF DEXA ($[M+1]^+$=393)

Fragment	HPLC-ESI-MS/MS					
	31,7		30,9		26,9	
	m/e	Int%	m/e	Int%	m/e	Int%
$[M+1]^+$	379,33	0	392,98	0	411,40	0
$[M+1]^+$-H_2O	361,13	25				
$[M+1]^+$-HF	359,22	100	372,99	100		
$[M+1]^+$-CH_2O					381,33	100
$[M+1]^+$-H_3FO	341,30	60	355,07	50		
$[M+1]^+$-H_4O_2					375,17	15
$[M+1]^+$-H_5FO_2	323,41	20	337,08	30		
$[M+1]^+$-CH_4O_2					363,13	40
$[M+1]^+$-H_6O_3					357,49	15
$[M+1]^+$-CH_6O_3					345,17	20
$[M+1]^+$-CH_5FO_2			325,05	10		
$[M+1]^+$-H_7FO_3			319,10	18		
$[M+1]^+$-CH_5FO_3	295,34	15	309,06	10		
$[M+1]^+$-CH_7FO_3					325,32	25
$[M+1]^+$-H_9FO_4			301,15	6		
$[M+1]^+$-$C_2H_7FO_3$	280,99	20				
$[M+1]^+$-CH_7FO_4	277,30	40	291,13	10		

Table S8. FRAGMENTATION OF PHOTOLYTIC PRODUCTS OF PRED ($[M+1]^+$=359)

Fragment	HPLC-ESI-MS/MS											
	**29,49**		**32,6**		**28,0**		**31,9**		**28,6**		**26,0**	
	m/e	Int%	m/e	Int%	m/e	Int%	m/e	Int%	m/e	Int%	m/e	Int%
$[M+1]^+$	358,80	1	299,01	1	317,39	0	373,38	0	375,13	0	377,07	2
$[M+1]^+$-H_2O	341,01	100	281,02	100	299,25	60	355,50	40	357,08	100	358,99	100
$[M+1]^+$-CO			271,02	60								
$[M+1]^+$-CH_2O											347,03	4
$[M+1]^+$-H_4O_2	323,08	44	263,11	68	281,35	40	337,43	10	339,01	80	341,06	14
$[M+1]^+$-CH_2O_2	313,04	46	253,02	40			327,43	45				
$[M+1]^+$-CH_4O_2					269,45	25					329,03	2
$[M+1]^+$-H_6O_3	305,01	30							321,09	42	323,08	4
$[M+1]^+$-$C_2H_2O_2$			240,93	2								
$[M+1]^+$-$C_2H_4O_2$									315,03	40	317,10	20
$[M+1]^+$-CH_4O_3	295,08	32					309,33	70	311,04	82		
$[M+1]^+$-CH_6O_3									308,94	4		
$[M+1]^+$-$C_2H_2O_3$							299,25	60				

$[M+1]^{+}$-H_8O_4									303,01	10		
$[M+1]^{+}$-$C_3H_6O_2$			225,09	16								
$[M+1]^{+}$-$C_2H_4O_3$			223,10	20								
$[M+1]^{+}$-$C_2H_6O_3$									297,03	12	299,09	4
$[M+1]^{+}$-$C_2H_{10}O_3$												
$[M+1]^{+}$-CH_6O_4	277,12	12							292,99	20		
$[M+1]^{+}$-$CH_{10}O_4$									288,99	10		
$[M+1]^{+}$-$C_2H_4O_4$	267,06	18					281,3	100	282,96	6		
$[M+1]^{+}$-$C_3H_8O_3$												
$[M+1]^{+}$-$C_2H_6O_4$	265,19	12										
$[M+1]^{+}$-$C_2H_8O_4$									279,11	30		
$[M+1]^{+}$-$C_5H_6O_2$					219,45	100						
$[M+1]^{+}$-$C_3H_6O_4$	253,15	8										
$[M+1]^{+}$-$C_4H_8O_4$	237,16	12										

Table S9. FRAGMENTATION OF PHOTOLYTIC PRODUCTS OF PREDLO ($[M+1]^{+}$361)

Fragment	HPLC-ESI-MS/MS									
	**28.8**		**31.6**		**26.8**		**27.9**		**24.7**	
	m/e	Int%	m/e	Int%	m/e	Int%	m/e	Int%	m/e	Int%
$[M+1]^{+}$	361,01	1	301,01	1	375,25	0	377,33	0	379,36	0
$[M+1]^{+}$-H_2O	342,99	100	282,96	100	357,60	55	359,32	80	361,43	100
$[M+1]^{+}$-H_4O_2	325,00	50	265,18	10	339,35	40	342,20	100		
$[M+1]^{+}$-H_6O_3	307,03	40	246,97	5	321,71	10	323,53	55		
$[M+1]^{+}$-CH_5O_2									331,24	25
$[M+1]^{+}$-CH_4O_3	297,02	8			311,27	45	313,46	10		
$[M+1]^{+}$-CH_7O_3									313,35	30
$[M+1]^{+}$-H_8O_4	289,11	10			303,23	10	305,82	40		
$[M+1]^{+}$-CH_6O_4	279,08	12			293,54	45	295,63	75		
$[M+1]^{+}$-$C_2H_8O_4$	265,08	10								

Table S10. FRAGMENTATION OF PHOTOLYTIC PRODUCTS OF TRIAM ($[M+1]^{+}$=435)

Fragment	HPLC-ESI-MS/MS									
	34,9		32,2		30,9		34,0		28,0	
	m/e	Int%	m/e	Int%	m/e	Int%	m/e	Int%	m/e	Int%
$[M+1]^{+}$	391,47	0	434,86	1	451,47	0	465,04		453,34	0
$[M+1]^{+}$-H_2O									435,21	40
$[M+1]^{+}$-HF			415,01	100	431,42	30	444,98			
$[M+1]^{+}$-H_4O_2									418,48	20

$[M+1]^+$-H_3FO			397,10	38	413,32	70	427,12			
$[M+1]^+$-CH_5FO							413,00			
$[M+1]^+$-H_7FO_2					395,39	100				
$[M+1]^+$-C_3H_6O			377,13	2						
$[M+1]^+$-$C_2H_2O_2$									395,48	80
$[M+1]^+$-CH_7FO_2							394,94			
$[M+1]^+$-C_2HFO_2									377,28	20
$[M+1]^+$-C_3H_7FO			357,03	30						
$[M+1]^+$-$C_3H_9FO_2$			339,02	30	355,33					
$[M+1]^+$-$C_2H_5FO_3$	295,33	70					368,83			
$[M+1]^+$-$C_2H_7FO_4$							350,96			
$[M+1]^+$-$C_3H_{11}FO_3$			321,04	12						
$[M+1]^+$-$C_3H_5FO_4$	267,27	100								
$[M+1]^+$-$C_3H_9FO_4$							336,88			
$[M+1]^+$-$C_5H_{13}FO_2$			311,08	10						
$[M+1]^+$-$C_3H_{11}FO_5$							319,02			
$[M+1]^+$-$C_5H_{15}FO_3$			293,07	10						
$[M+1]^+$-$C_5H_{13}FO_4$							308,93			
$[M+1]^+$-$C_6H_{17}FO_3$			279,04	6						
$[M+1]^+$-$C_7H_{19}FO_3$			265,09	2						

Figure S1. Tap water solutions fortified with 50 µg L^{-1} of each GCs and kept in the dark at room temperature for a monitoring period of 3 hours for PRED (☐) and PREDLO (+), 5 hours for BETA (×), DEXA (Δ) and TRIAM(○), 16 hours for CORT (◊) and HCORT(✳).

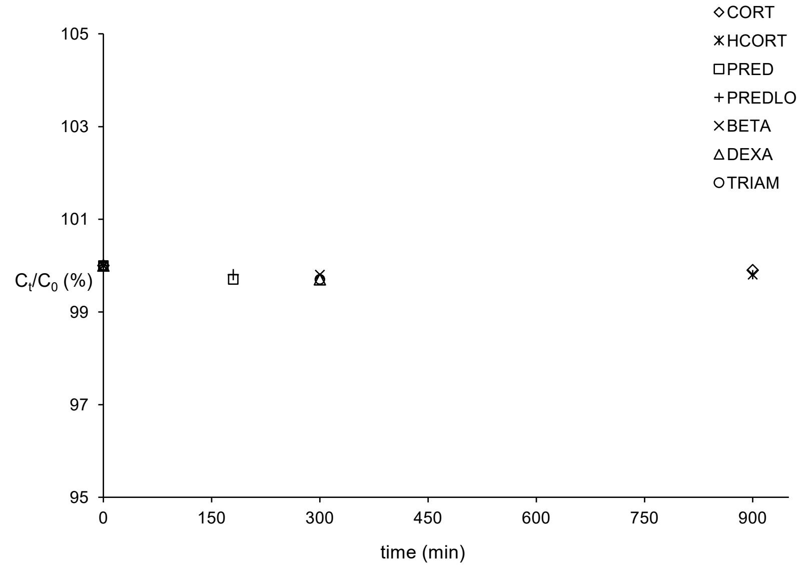

Figure S2. HPLC-UV chromatogram of BETA (black line) in presence of the maximum amount of photoproducts (red line) (a); photodegradation profile of BETA and evolution profile of the generated photoproducts verified by HPLC-UV (b) (90 minutes irradiation).

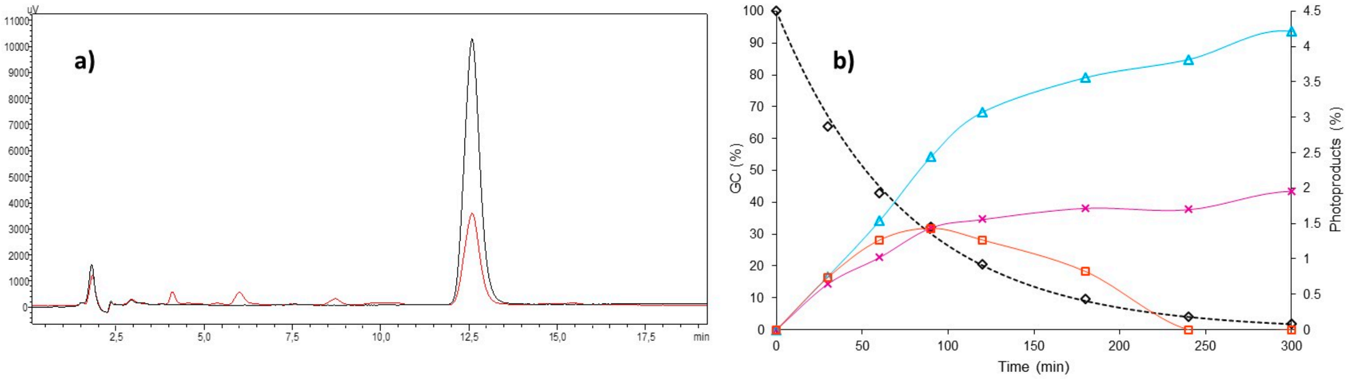

Supplement: Supplementary file 1 [file ijerph-17-08717-s001.pdf]
